# Naringenin Regulates Doxorubicin-Induced Liver Dysfunction: Impact on Oxidative Stress and Inflammation

**DOI:** 10.3390/plants9040550

**Published:** 2020-04-24

**Authors:** Adil Farooq Wali, Summya Rashid, Shahzada Mudasir Rashid, Mushtaq Ahmad Ansari, Mohammad Rashid Khan, Nazrul Haq, Dhafer Yahya Alhareth, Ajaz Ahmad, Muneeb U. Rehman

**Affiliations:** 1 RAK College of Pharmaceutical Sciences, RAK Medical and Health Sciences University, Ras Al Khaimah 11172, UAE; farooq@rakmhsu.ac.ae; 2 Department of Pharmacology & Toxicology, College of Pharmacy Girls Section, Prince Sattam Bin Abdulaziz University, P.O. Box 173, Al-Kharj 11942, Saudi Arabia; s.abdulrashid@psau.edu.sa; 3 Division of Veterinary Biochemistry, Faculty of Veterinary Science and Animal Husbandry, SKUAST-Kashmir, Shuhama, J&K 190006, India; drsmrashid786@gmail.com; 4 Department of Pharmacology & Toxicology, College of Pharmacy, King Saud University, P.O. Box 2457, Riyadh 11451, Saudi Arabia; muansari@ksu.edu.sa (M.A.A.); kmohammad@ksu.edu.sa (M.R.K.); 437105645@ksu.edu.sa (D.Y.A.); 5 Department of Pharmaceutics, College of Pharmacy, King Saud University, P.O. Box 2457, Riyadh 11451, Saudi Arabia; nhaq@ksu.edu.sa; 6 Department of Clinical Pharmacy, College of Pharmacy, King Saud University, P.O. Box 2457, Riyadh 11451, Saudi Arabia

**Keywords:** doxorubicin, naringenin, NF-κB, reactive oxygen species, hepatotoxicity

## Abstract

Doxorubicin (Dox) is an operational and largely used anticancer drug, used to treat an array of malignancies. Nonetheless, its beneficial use is constrained due to its renal and hepatotoxicity dose dependently. Numerous research findings favor the use of antioxidants may impact Dox-induced liver injury/damage. In the current study, Wistar rats were given naringenin (50 and 100 mg/kg b.wt.) orally for 20 days as prophylactic dose, against the hepatotoxicity induced by single intraperitoneal injection of Dox (20 mg/kg b.wt.). Potency of naringenin against the liver damage caused by Dox was assessed by measuring malonyl aldehyde (MDA) as a by-product of lipid peroxidation, biochemical estimation of antioxidant enzyme system, reactive oxygen species (ROS) level, and inflammatory mediators. Naringenin-attenuated ROS production, ROS-induced lipid peroxidation, and replenished reduced antioxidant armory, namely, catalase (CAT), glutathione reductase (GR), superoxide dismutase (SOD), glutathione peroxidase (GPx), and glutathione (GSH). Naringenin similarly diminished expression of Cox-2 and levels of NF-κB and other inflammatory molecules induced by the Dox treatment. Histology added further evidence to the defensive effects of naringenin on Dox-induced liver damage. The outcomes of the current study reveal that oxidative stress and inflammation are meticulously linked with Dox-triggered damage, and naringenin illustrates the potential effect on Dox-induced hepatotoxicity probably through diminishing the oxidative stress and inflammation.

## 1. Introduction

Doxorubicin (Dox) is an antineoplastic drug which is used for the treatment of various human malignancies especially solid tumors. Its success is attributed to exceptional efficacy and widespread beneficial effects. However, its use is associated with large number of acute and chronic dose-related side effects and large-scale toxicities, e.g., intestinal tract epithelium dysfunctioning, bone marrow degeneration, hepatotoxicity, cardiotoxicity, and nephrotoxicity [1,2]. The main organ responsible for breakdown and elimination of chemicals is liver which gets targeted by Dox use. Hepatic insult contributes majorly to Dox toxicity and in 40% of cases, hepatic toxicity was observed [3,4]. This liver insult may possibly be by cellular oxidative stress, interfering in the mitochondrial electron transport chain (ETC) and apoptosis.

Dox has been reported to hamper biological activities within the cell. Nonetheless, exact molecular pathology of toxicity of Dox is not fully understood yet. In liver, cytochrome family enzymes and cytoplasmic reductase metabolize Dox to the doxorubicinol and other aglycone metabolites, toxic to liver [5]. Many research groups have reported Dox to elevate production of reactive oxygen species (ROS) and reactive nitrogen species (RNS) that play role in number of cellular mechanisms like redox cycling of quinine moiety of Dox and disrupt iron homeostasis [6]. 

The Dox redox cycling starts from one-electron redox reaction which gives rise to the formation of “Dox semiquinone” from “Dox quinone” by oxidation. Semiquinone can be changed to its inherent “Dox quinone” form in the presence of oxygen, producing superoxide (O^2−^) as an end product [7,8]. Superoxide (O^2−^) anions have potential to damage biological components and can also be converted to highly reactive ROS/RNS [9].

Cell membranes of hepatocytes are vulnerable to Dox-induced free radical injury. Therefore, peroxidation continues autocatalytically leading to functional and structural abnormalities. Dox-induced oxidative stress causes serious disturbances in the synthesis of DNA, RNA (DNA-dependent), and proteins, which in turn hamper regenerative potential of the organelles. Such irreversible phenomenon leads to huge elevation of hepatic enzymes and necrosis or apoptosis of hepatocytes [10]. 

Increased ROS and RNS and exhaustion of antioxidants endogenously by Dox activates massive immune reaction [11]. Inflammation is a complex process triggered by activation of different types of immune cells [12]. Many reports demonstrate elevated inflammatory mediators like inflammatory cell infiltration, suggesting role of inflammation in Dox-induced organ damage [13,14]. NF-κB is documented as an imperative redox-sensitive transcriptional factor regulating coding of genes for chemokines, inflammatory cytokines, and adhesion factors. TNF-α is another crucial marker in pro-inflammatory cytokines/chemokines network activated by Dox [6,15]. Interleukin-6 (IL-6) is a significant molecule like TNF-α in liver response [16,17]. Cox-2 is yet another important factor responsible for instigating inflammatory cascade. Under normal physiological conditions, Cox-2 is slightly present in tissues, whereas in inflammatory reactions, Cox-2 shoots up triggering prostaglandin synthesis, which further plays a part in pathological processes. In Dox toxicity, overexpressed Cox-2 and associated prostaglandins interfere with protein breakdown, which aggravates ROS in liver and facilitates triggering of inflammatory cytokines exacerbating hepatic injury [18]. Inhibition of NF-κB, Cox-2, and PGE-2 prevents activation of downstream cytokine network and provides protection against Dox-induced hepatotoxicity. 

Published reports have suggested plant compounds having antioxidant and anti-inflammatory potential to mitigate Dox damage. Fullerenol [2], quercetin [19], chrysin [11], silybin [20], D-Limonene [6], sylimarin [21], resveratrol [22], etc. have been found to protect against Dox-induced nephrotoxicity. Naringenin (4’,5,7-trihydroxyflavanone) is a main flavonone abundantly found in citrus fruits like lemons, tangerines, etc. [23]. Naringenin has been used as an important ingredient in traditional Chinese medicine [24,25]. It has been observed to have exceptional pharmacological properties ranging from antioxidant to anticancer [2,26,27]. 

The objective of our work was to evaluate the potential of naringenin in the prevention of hepatic injury induced by Dox in Wistar rats. Therefore, estimation of ROS, LPO, antioxidant armory, levels of cytokines, and inflammatory mediators as well as structural examination of the liver were undertaken.

## 2. Materials and Methods

### 2.1. Chemicals and Reagents

Doxorubicin, naringenin, and other chemicals of highest purity grades were purchased from Sigma-Aldrich, (Saint Louis, MO, USA). 

### 2.2. Animals and Experimental Design

All the animal experiments were conducted in accordance with animal ethics guidelines and approved by the Institutional Ethics Committee. Four to six-week-old male albino Wistar rats (170–200 g) were used for the present study. They were housed in controlled environment under standard conditions of temperature and humidity with an alternating 12 h light and dark cycle. The animals were fed *ad libitum* food and water. Twenty-four male adult Wistar rats (*n* = 24) were randomly divided into four groups of six rats each. After adaptation period of 1 week, group I animals were given vehicle orally for 20 days. Naringenin was given orally daily at two doses, 50 and 100 mg/kg b.wt. to groups III and IV animals, respectively, for 20 days (Table 1). A single intraperitoneal injection of Dox of 20 mg/kg body weight dose was given to groups II, III, and IV animals on 20th day [28,29,30,31]. After 24 h of Dox administration, rats were sacrificed by cervical dislocation under mild anesthesia using ketamine/xylazine cocktail (KX rat cocktail 0.1 mL/100 g rat wt. IP having 91 mg/kg ketamine and 9.1 mg/kg xylazine). Liver samples were taken at the same time to do further processing by immunohistochemistry, biochemical estimations, and histological analysis. There was 100% survival of animals in all the groups.

### 2.3. Post Mitochondrial Supernatant Preparation

Livers were cleaned and immediately perfused with cold saline. In chilled phosphate buffer (0.1 M, pH 7.4), the livers were homogenized in a homogenizer. To separate the nuclear debris, the homogenate was centrifuged at 700 g for 10 min in cooling centrifuge. The post mitochondrial supernatant (PMS) in the form of aliquot so obtained was used as a repertoire of various enzymes [6].

### 2.4. Estimation of Serum Enzyme Activities

#### 2.4.1. Estimation of Serum Aspartate Aminotransferase (AST) and Alanine Aminotransferases (ALT) 

AST and ALT activities were determined by the method of Reitman and Frankel [32]. Each substrate (0.5 mL; 2 mM a-ketoglutarate and either 200 mM L-alanine or L-aspartate) was incubated for 5 min at 37 °C in a water bath. Serum (0.1 mL) was then added, and the volume was adjusted to 1.0 mL with 0.1 M (pH 7.4) phosphate buffer. The reaction mixture was incubated for exactly 30 and 60 min at 37 °C for ALT and AST, respectively. Then, 0.5 mL of 1 mM dinitrophenyl hydrazine (DNPH) was added to the reaction mixture; after another 30 min at room temperature, the color was developed by the addition of 5.0 mL of NaOH (0.4 N), and the product was read at 505 nm.

#### 2.4.2. Assay for Lactate Dehydrogenase Activity

Lactate dehydrogenase (LDH) activity was estimated in serum by the method of Kornberg [33]. The assay mixture consisted of 0.2 mL of serum, 0.1 mL of 0.02 M-NADH, 0.1 mL of 0.01 M sodium pyruvate, 1.1 mL of 0.1 M (pH 7.4) phosphate buffer, and distilled water, in a total volume of 3 mL. Enzyme activity was recorded at 340 nm, and the activity was calculated as nmol NADH oxidized/min per mg protein.

#### 2.4.3. Estimation of Protein

The protein concentration was determined by the method of Lowry et al. [34] using BSA (Bovine serum albumin) as standard.

#### 2.4.4. Estimation of Alkaline Phosphatase (ALP)

ALP estimation was done by a commercially available kit (Accurex Biomedical Private Limited, Mumbai, India).

### 2.5. Measurement of ROS

ROS were evaluated as described by Liua et al. [35] on the basis of oxidation of 2’7’-dichlorodihydrofluorescein diacetate to 2’7’-dichlorofluorescein. Briefly, the homogenate and chilled Locke’s buffer (154 mM NaCl, 5.6 mM KCl, 3.6 mM NaHCO_3_, 2.0 mM CaCl2, 10 mM glucose, and 5 mM HEPES, pH 7.4) were mixed in the ratio of 1:20 to a attain concentration of 5 mg tissue/mL. The reaction mixture (1 mL) composed of Locke’s buffer (pH 7.4), 0.2 mL homogenate, and 10 mL of DCFH-DA (5 mM) was kept for 45 min at room temperature. The conversion of DCFH-DA to the fluorescent product DCF was measured using a spectrofluorimeter with excitation at 484 nm and emission at 530 nm. ROS formation was quantified from a DCF standard curve, and the data are expressed as pmol DCF formed/min/mg protein.

### 2.6. Estimation of Lipid Peroxidation

The estimation for microsomal lipid peroxidation was done following the method of Wright et al. [36]. The reaction mixture consisted of 0.58 mL phosphate buffer (0.1 M, pH 7.4), 0.2 mL microsome, 0.2 mL ascorbic acid (100 mM), and 0.02 mL ferric chloride (100 mM), in a total volume of 1 mL. This reaction mixture was then incubated at 37 °C in a shaking water bath for 1 h. The reaction was stopped by the addition of 1 mL trichloroacetic acid (10%). Following the addition of 1.0 mL thiobarbituric acid (TBA) (0.67%), all the tubes were placed in a boiling water bath for a period of 20 min. The tubes were shifted to an ice bath and then centrifuged at 2500× *g* for 10 min. The amount of MDA formed in each of the samples was assessed by measuring the optical density of the supernatant at 535 nm. The results were expressed as nmol TBA formed/h per g tissue at 37 °C by using a molar extinction coefficient of 1.56 × 10^5^ M^−1^ cm^−1^.

### 2.7. Estimation of Antioxidant Enzyme Armory

#### 2.7.1. Assay for Superoxide Dismutase Activity (SOD)

SOD activity was measured by the method of Marklund and Marklund [37]. The reaction mixture consisted of 2.875 mL Tris–HCl buffer (50 mM, pH 8.5), pyrogallol (24 mM in 10 mM-HCl), and 0.1 mL PMS, in a total volume of 3 mL. Enzyme activity was measured at 420 nm and was expressed as units/mg protein. One unit of enzyme is defined as the enzyme activity that inhibits the auto-oxidation of pyrogallol by 50%.

#### 2.7.2. Catalase Activity

CAT activity was determined by the method of Claiborne [38]. The reaction mixture consisted of 1.95 mL phosphate buffer (0.1 M, pH 7.4), 1.0 mL hydrogen peroxide (0.10 mM), and 0.05 mL 10% PMS in a final volume of 3 mL. Changes in absorbance were recorded at 240 nm. Catalase activity was calculated as nmol H_2_O_2_ consumed/min/mg protein.

#### 2.7.3. Estimation of Glutathione (GSH)

GSH was assessed by the method described by Rashid et al. [14]. A quantity of 1.0 mL of 10% PMS mixed with 1.0 mL of (4%) sulphosalicylic acid was taken, incubated at 4 °C for a minimum period of 1 h and then centrifuged at 4 °C at 1200× *g* for 15 min. The reaction mixture of 3.0 mL was composed of 0.4 mL of supernatant, 2.2 mL phosphate buffer (0.1 M, pH 7.4), and 0.4 mL dithio-bis-2-nitrobenzoic acid (4 mg/mL). The yellow color developed was read immediately at 412 nm on the spectrophotometer. GSH concentration was calculated as nmol GSH conjugates/g tissue.

#### 2.7.4. Glutathione Reductase (GR) Activity

GR activity was measured by the method described by Rashid et al. [14]. The reaction mixture consisted of 1.65 mL phosphate buffer (0.1 M, pH 7.6), 0.1 mL EDTA (0.5 mM), 0.05 mL GSH (1 mM), 0.1 mL NADPH (0.1 mM), and 0.1 mL of 10% PMS, in a total volume of 2 mL. Enzyme activity was quantified at 25 °C by measuring the disappearance of NADPH at 340 nm and was calculated as nmol NADPH oxidized/min per mg protein using a molar extinction coefficient of 6.22 × 10^3^/M per cm.

#### 2.7.5. Glutathione Peroxidase Activity

The activity of GPx was calculated by the method of Mohandas et al. [39]. The total volume of 2 mL was composed of 0.1 mL EDTA (1 mM), 0.1 mL sodium azide (1 mM), 1.44 mL phosphate buffer (0.1 M, pH 7.4), 0.05 mL glutathione reductase (1 IU/mL is equivalent to 1 mol Oxidised glutathione (GSSG) reduced/min per mL), 0.05 mL GSH (1 mM), 0.1 mL NADPH (0.2 mM), 0.01 mL H_2_O_2_ (0.25 mM), and 0.1 mL of 10% PMS. The depletion of NADPH at 340 nm was recorded at 25 °C. Enzyme activity was calculated as nmol NADPH oxidized/min per mg protein with a molar extinction coefficient of 6.22 × 10^3^/M per cm.

### 2.8. Measurement of Nitric Oxide (NO)

Production of NO was evaluated by the method of Green et al. [40]. Different concentrations of naringenin in standard phosphate buffer solution (pH 7.4) were incubated with an equal volume of sodium nitroprusside solution (5 mM) in standard phosphate buffer (pH 7.4) at 25 °C for 5 h. In an identical manner, solutions of different concentrations of ascorbic acid (25–400 μg/mL) in standard phosphate buffer (pH 7.4) were also incubated with an equal volume of sodium nitroprusside solution (5 mM) in standard phosphate buffer (pH 7.4). Controlled experiments without the test compound but with equivalent amount of buffer were also conducted. After incubation, 0.5 mL of the incubation mixture was mixed with 0.5 mL of Griess’ reagent (sulphanilamide 1%, *o*-phosphoric acid 2%, and naphthyl ethylene diamine dihydrochloride 0.1%), and the absorbance was measured at 546 nm. From the absorbance, the % scavenging activity was calculated using the above formula. The experiments were performed in triplicates.

### 2.9. Assay for Hydrogen Peroxide (H_2_O_2_)

Hydrogen peroxide (H_2_O_2_ standard curve) was assayed by the method of Pick and Keisari [41]. 

### 2.10. Histopathological Examination

The liver was quickly removed and preserved in 10% neutral buffered formalin for histological processing. The liver tissue was longitudinally sectioned with a microtome after embedding in paraffin wax. Hematoxylin and eosin staining were used to stain the tissue, and the tissue was observed under the microscope.

### 2.11. Immunostaining

To observe the protective effects of naringenin on DOX-induced inflammation in liver tissue, it was assessed by immunohistochemistry. Sections of formalin-fixed, paraffin-embedded livers were obtained on poly-L-lysine-coated slides. The method was done as described by Rashid et al. [14]. Sections were deparaffinized in xylene and then rehydrated through a graded alcohol series. Antigen retrieval was performed by incubating slides in citrate buffer (pH 6.0) (10 mM) at 95 °C for 20 min. Endogenous peroxidase activity was blocked with 3% H_2_O_2_ for 30 min. To detect Cox-2, sections were incubated overnight at 4 °C with anti-cox-2 under humid conditions (1:400; Santa Cruz Biotechnology, Inc., Dallas, Texas, USA). Next day, the slides were washed three times in Tris buffers (pH-6.0) and were incubated with a biotinylated Goat Anti-Polyvalent Plus (Thermo Fisher Scientific, Waltham, MA, USA) for 30 min at room temperature. This step was followed by further washing in Tris buffer and incubation of slides at room temperature with Streptavidin Peroxidase Plus (Thermo Fisher Scientific, Waltham, MA, USA) that binds to the biotin present on the secondary antibody. After washing in Tris buffer, the immunostaining reaction product was developed using 3,30-diaminobenzidine (DAB Plus substrate, Thermo Fisher Scientific, Waltham, MA, USA). After immunoreactivity, slides were dipped in distilled water, counterstained with Harris hematoxylin and dried, and finally, the sections were mounted with DPX and covered with cover slips. The slides were ready to be observed under microscope.

### 2.12. Inflammatory Mediators

Inflammatory mediators, NF-κB (Cat No: LS-F69373, LSBio Inc. WA, USA), TNF-α (Cat No: Cat # 88-7340-22, Invitrogen, Thermo Fischer, USA), IL-6 (Cat No: 670.010.192, Diaclone SAS, France), IL-1β (Cat No: 670.040.096, Diaclone SAS, France), TGF-β (Cat No: 670 020, Diaclone SAS, France), and PGE-2 (Cat No: MBS3808784, MyBioSource, Inc, San Diego, CA, USA), were analyzed by ELISA-based commercially available kits.

### 2.13. Ethical Statement

All the procedures for using experimental animals were checked, and proper permission was obtained from the Institute’s animal ethics committee (Approval No: RAKMHSU-REC-08-2019-F-P).

### 2.14. Statistical Analysis

The data was subjected to statistical analysis by using appropriate software like SPSS 20.0. All the results are presented as means ± SE. Differences between groups were analyzed using analysis of variance (ANOVA) followed by Tukey–Kramer multiple comparisons test, and minimum criterion for statistical significance was set at *p* < 0.05 for all comparisons.

## 3. Results

### 3.1. Effect of Naringenin Treatment on the ROS Levels

**Figure 1:**
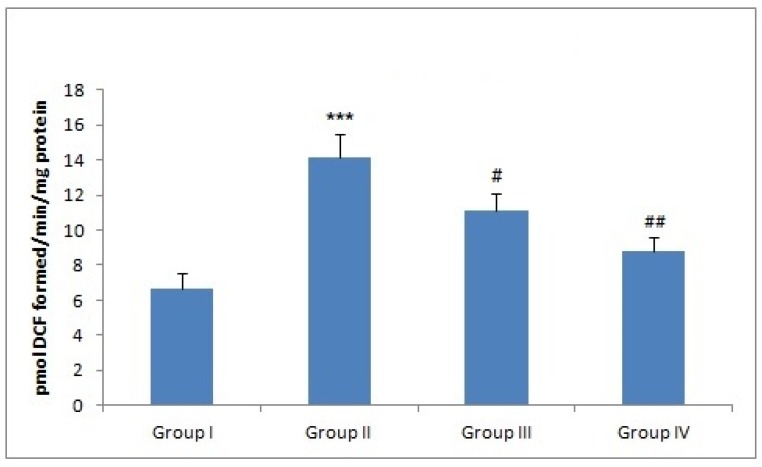
Effect of naringenin and doxorubicin treatment on ROS levels. In Dox-treated group-II, the reactive oxygen species (ROS) level was increased significantly (*** *p* < 0.001) as compared to the control group -I. Treatment with naringenin (50 and 100 mg/kg b. wt.) significantly attenuated ROS levels in group III (^#^ *p* < 0.05) and group IV (^###^ *p* < 0.01) as compared to group II.

ROS were significantly higher (*** *p* < 0.001) in group II (Dox-treated group) when compared with the control group, revealing that Dox increases oxidative stress by instigating ROS. Naringenin supplementation decreased ROS level in the liver tissue in both group III (^#^ *p* < 0.05) and group IV animals (^###^ *p* < 0.001) by scavenging free radicals (Figure 1).

### 3.2. Effect of Naringenin Treatment on the Antioxidant Enzyme System

The effect of naringenin treatment on Dox-induced exhaustion of different antioxidants was studied. The results are shown in Table 2. There was a significant difference (*** *p* < 0.001) in the activity of antioxidants between control and only Dox-treated group. However, naringenin treatment restored back the activity of all antioxidant enzymes to normal (Table 2).

### 3.3. Effect of Naringenin Treatment on H_2_O_2_ Levels

There was a significant increase in H_2_O_2_ levels in the liver tissue in group II (*** *p* <0.001), when compared with group I. Naringenin treatment to groups III and IV caused marked reduction of H_2_O_2_ at both the doses (Table 2). 

### 3.4. Effect of Naringenin Treatment on MDA Levels

**Figure 2:**
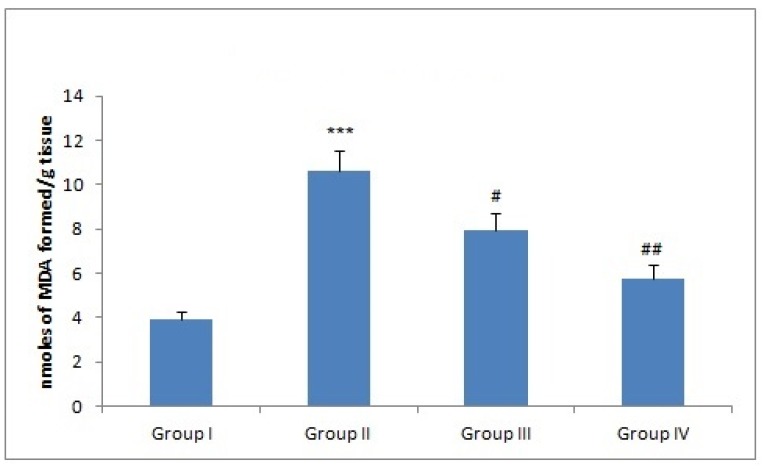
Effect of naringenin and doxorubicin treatment on malonyl aldehyde (MDA) levels. In Dox-treated group-II, the MDA level was increased significantly (*** *p* < 0.001) as compared to the control group-I. Treatment with naringenin (50 and 100 mg/kg b. wt.) significantly attenuated MDA levels in group III (^#^ *p* < 0.05) and group IV (^###^ *p* < 0.01) as compared to group II.

MDA is a well-known biomarker of oxidative stress. There was a sharp increase in MDA in Dox-administered group II as compared to group I (*** *p* < 0.001). However, naringenin treatment at both the doses attenuated MDA levels in group III (^#^ *p* < 0.005) and group IV (^##^ *p* < 0.01) (Figure 2). 

### 3.5. Naringenin Treatment Alleviates Serum Toxicity Parameters in Dox-Induced Liver Toxicity

There was a significant increase in liver toxicity markers found in serum of Dox-treated group II animals as compared to group I (*** *p* < 0.001). Naringenin treatment alleviated the levels of liver toxicity markers when compared with Dox-treated group at both the doses significantly (Table 3). 

### 3.6. Effect of Naringenin and Dox Treatment on NF-κB and Other Inflammatory Mediators

There was an increase in NFkB levels in group II when compared with group I (*** *p* < 0.001). However, naringenin treatment to group III and group IV animals decreased the levels of NFkB significantly and dose dependently. Also, levels of pro-inflammatory cytokines like TNF-α, IL-1β, PGE-2, TGF-β, and IL-6 were increased significantly in Dox-treated group II in comparison to untreated control group I (*** *p* < 0.001). However, naringenin treatment to groups III and IV attenuated TNF-α, IL-1β, PGE-2, TGF-β, and IL-6 levels significantly at both the doses (^#^ *p* < 0.05, ^##^ *p* < 0.01, and ^###^ *p* < 0.001, respectively; Table 4).

### 3.7. Effect of Naringenin on NO Production

**Figure 3:**
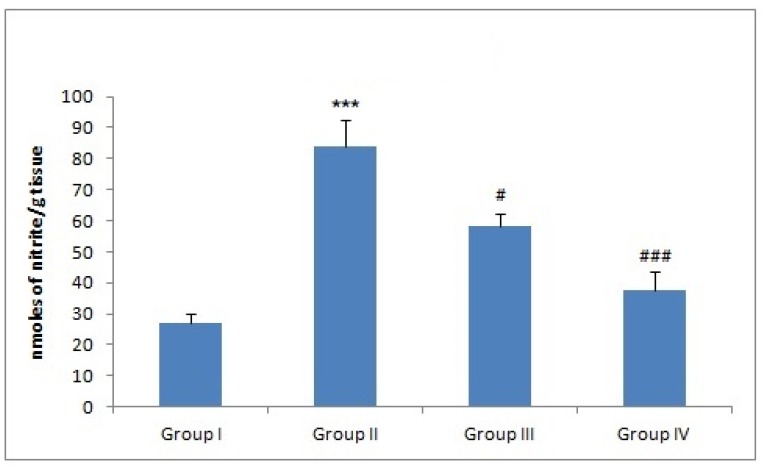
Effect of naringenin and doxorubicin on nitrite levels. In Dox-treated group-II, the nitrite levels were significantly increased (*** *p* < 0.001) as compared to the control group-I. Treatment with naringenin significantly (50 and 100 mg/kg b. wt.) attenuated nitrite levels in group III (^#^ *p* < 0.05) and group IV (^###^ *p* < 0.001) as compared to group-II.

There was a significant hepatic NO production in group II as compared to group I animals (*** *p* < 0.001). However, treatment with naringenin effectively reduced NO formation in groups III and IV (^##^ *p* < 0.01 and ^###^ *p* < 0.001) when compared with group II (Figure 3).

### 3.8. Effect of Naringenin on Liver Histology

**Figure 4:**
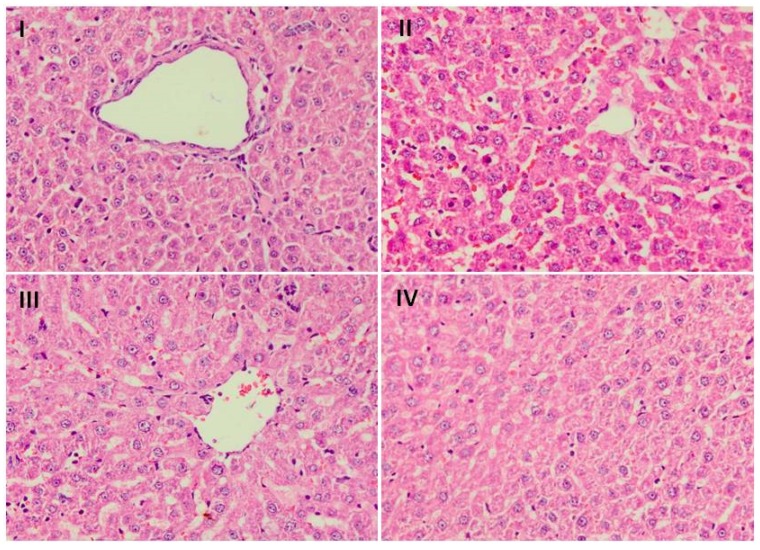
Effect of naringenin treatment on Dox-induced pathological changes in liver. Photomicrographs of H&E staining of histological sections of liver depicting different experimental groups, group I indicate normal histoarchitecture of liver sections. Group II shows extensive disintegration of normal architecture in the Dox-treated group. In groups III and IV, naringenin treatment showed protection against naringenin-induced pathological changes. Both the doses of naringenin maintained the integrity of central vein and hepatocytes. Magnification: 40×.

Group I histology figures revealed normal histoarchitecture of liver tissue (Figure 4). Administration of Dox led to infiltration of cells, dilatation of sinusoids, degeneration of hepatocytes, periportal fibrosis, focal necrosis, and steatosis, and hence, tissue injury/atrophy in group II. However, there was less restoration of histological architecture by naringenin supplementation in group III. Group IV showed complete restoration of normal histoarchitecture of liver tissue by naringenin supplementation at higher dose.

### 3.9. Effect of Naringenin on Expression of COX-2

**Figure 5:**
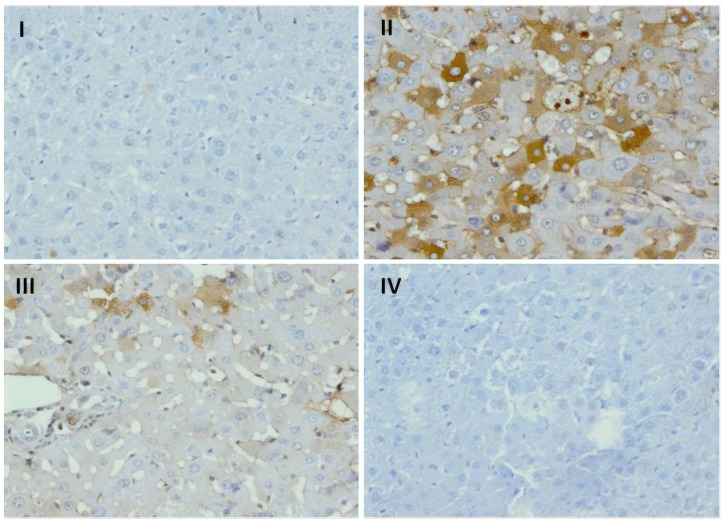
Effect of naringenin treatment on Cox-2 expression. Photomicrographs of hepatic sections depicting immunohistochemical analyses, brown color indicates specific immunostaining of Cox-2 and light blue color indicates counter staining by nuclear hematoxylin. The hepatic section of Dox-treated group II has more Cox-2 immunopositive staining as indicated by brown color as compared to the control group I, whereas treatment of naringenin (50 and 100 mg/kg b. wt.) in groups III and IV reduced Cox-2 immunostaining as compared to group II. Original magnification: 40×.

The immunohistochemical slides show strong expression of Cox-2 in hepatocytes in rats subjected to DOX-treatment when compared with group I, where no significant staining of Cox-2 was observed (Figure 5). There was decreased cytoplasmic staining of Cox-2 in hepatic cells in naringenin (50 mg/kg b.wt.)-supplemented group as compared to group II animals. Moreover, naringenin (100 mg/kg b.wt.)-treated rats at higher dose showed least or negligible staining of Cox-2 as compared to DOX-treated rats which infers anti-inflammatory potential of naringenin.

## 4. Discussion

Dox-induced hepatic toxicity is well documented, which may be attributed to the production of ROS instigating oxidative stress besides inflammation via release of inflammatory cytokines [6,42]. The natural compounds are gaining much attention across the world due to their antioxidant properties [43]. Our work is designed to evaluate the antioxidant and anti-inflammatory properties of naringenin in Dox-associated hepatic damage in Wistar rats. Administration of Dox is a source of adverse effects like genotoxicity, nephrotoxicity, hepatotoxicity, and cardiotoxicity, which restricts its clinical use [16]. Researchers are trying to investigate compounds that can mitigate Dox-induced hepatoxicity.

The mechanism of action involves either Dox semiquinone formation with the help of NADPH-dependent reductase enzyme pathway by one-electron reduction of Dox, which leads to the formation of superoxide ions, which then react with iron in a nonenzymatic process, generates H_2_O_2_ by delocalizing Fe^2+^ from ferritin resulting in oxidative stress [7,44]. Phytochemicals like resveratrol, quercetin, and anthocyanins [28,45,46,47] with tremendous antioxidant potential have been reported to counter increase ROS levels [27,48]. Naringenin is classified under the category of flavonone and has been reported to have high antioxidant activity compared to the other flavonoids, e.g., anthocyanins, flavones, etc. [49]. Similarly, we show that administration of naringenin reduced Dox-induced ROS generation in liver of Wistar rats which is further supported by the previous studies of Rehman et al. and Koyuncu et al. (Figure 1) [27,48]. Lipid peroxidation involves oxidation of lipids, yields an aldehyde (MDA). Administration of Dox has been closely associated with lipid peroxidation which is attributed to increase in free radical formation, thereby, signifying a state of oxidative injury as reported previously by Aluise et al. and Kuzu et al. in the liver and kidneys of animals after Dox administration [6,11,50]. The decrease in MDA levels in Dox-treated groups followed by administration of naringenin indicates the action of naringenin as a potent antioxidant and free radical scavenger (Figure 2). The similar effect of naringenin was reported by Subburaman, having extraordinary antioxidative and free radical scavenging activity [51]. Naringenin diminishes membrane fluidity remarkably. Membrane fluidity is the freedom of motion of lipid molecules relatively in lipid membrane. Naringenin mediates lipid packing order to reduce membrane fluidity by accumulating in the hydrophobic core of the membrane. Henceforth, it increases membrane rigidity and decreases the association between free radicals and lipids resulting in alleviation of lipid peroxidation [52].

Doxorubicin is an anthracycline antibiotic, whose mechanism of action is by alteration of membrane function, free radical formation, and intercalation of DNA [53]. In the current work, marked liver damage in Dox-treated group was seen as there was an elevation of liver markers (AST and ALT) in the Dox-treated groups signifying the dose at which the drug is given, being hepatotoxic. When antioxidant system fails and oxidative stress/ROS generation is continuous, hepatoxicity is evident. The increased biomarker level in serum in the present study is validation of liver impairment that may be secondary event following drug-induced hepatic toxicity with enzyme leakage from the liver cells. Likewise, Zhao, Henninger et al., and Afsar et al. had shown altered serum AST and ALT levels contributing to the liver toxicity by Dox [54,55,56]. Our findings revealed the hepatoprotective role of naringenin in rats which underwent Dox-induced hepatic damage followed by its reversal with the administration of naringenin (Table 3) by shielding hepatocytes against necrosis, cholestasis, and membrane permeation making our findings in agreement with the findings of Hernández-Aquino et al. and Osama et al. [52,57].

In the present study, we separately assessed ROS as well as individual markers of cellular antioxidant defense system including GSH, GSSG, CAT, SOD, GPx, GR, and H_2_O_2_ levels. Lipid peroxidation is the hallmark of oxidative stress. Lipid peroxidation results in increase of fibrogenic cytokines by stimulating the formation of collagen and liver stellate cells. Dox administration results in a substantial increase in the lipid peroxidation as reported by Afsar et al., discovered by enhancing MDA concentration in liver, facilitating oxidative stress [54] as previously reported by Liu et al. [58]. The enhanced lipid peroxidation in the Dox-treated group may be due to GSH diminution and reduced antioxidants. Naringenin significantly protects liver against Dox-induced alteration in lipid peroxidation and membrane damage. This observation supports the indication that part of the mechanisms of hepatic injury in Dox-treated animals is linked with oxidative injury. The suppression of pro-oxidant activity and LPO results in Dox-induced hepatic damage, and the preclusion of this variation in rats treated with naringenin support the basis for using antioxidants to mitigate Dox-caused liver toxicity. This is in concurrence with the reports of Lee et al., and our results validate similar mechanism [59]. 

Superoxide dismutase is an essential enzyme that manages the dismutation of superoxides to hydrogen peroxide and oxygen, whereas catalase, another enzyme largely abundant in liver, governs conversion of hydrogen peroxide to water. Nevertheless, in GSH (glutathione) defense system, GSH oxidizes to GSSG with the help of GPx which successively is transformed to GSH via reduction reaction of GR, hence detoxifies toxic compounds to generate safer compounds [11,16]. Administration of Dox decreased the levels of antioxidant enzymes, namely, SOD, GPx, GR, and CAT in rats in response to free radical formation, supporting the serious role of oxidative stress in Dox hepatotoxicity. Kwatra et al., Rashid et al., and Song et al. [11,16,60] also observed oxidative damage in rats triggered by Dox due to the generation of H_2_O_2_, superoxide anion radicals, and other free radicals [61]. This results in the decreased ability of the liver to scavenge toxic hydrogen peroxide and hydroxyl and superoxide radicals. Coadministration of naringenin and Dox alleviated the biochemical changes induced by Dox in liver along with substantial increase in the level of hepatic antioxidant armory (Table 2). Our results simulate with the results reported by Han et al., Renugadevi et al., and Lee et al. [59,62,63]. These authors have observed the decrease in ROS and significant improvement in antioxidant enzymes under different conditions after the supplementation of naringenin. The results obtained infer the protective effect of naringenin at duo doses (50 mg/Kg and 100 mg/Kg b.wt) against hydrogen peroxide levels also. Oxidative stress leads to the formation of hydrogen peroxide. The antioxidative nature of naringenin reduces the formation of oxides and peroxides which is also well documented in earlier studies conducted by Rashmi et al. and Rehman et al. [27,64]. We obtained similar results in our current study.

In the present study, Dox administration progressed oxidative and nitrosative stress in liver. Dox administration enhanced NO may be by hypergeneration of superoxide radical anion, which may combine with NO forming peroxynitrite, a cytotoxic molecule which damages proteins, DNA, and ribonucleic acid. Reports show that peroxynitrite participate in nitrosative stress [65]. Therefore, the generation of nitrosative stress might be allied with peroxynitrite formation. The contribution of Dox in the oxidative stress and ROS and RNS generation is one of the mechanisms of action of Dox-caused liver damage as reported previously by Liu et al., Szwed, Mokini et al., Omobowale et al., and Mukhopadhyay et al. [6,17,58,66,67]. However, coadministration of naringenin strongly demonstrated potent antioxidant activity by attenuating oxidative stress markers, nitrosative stress, and replenishing the antioxidant status in liver (Figure 3). The experimental lessening in the activities of GR and GPx may be due to ROS generation by Dox, thereby increasing the growth of toxic metabolites of Dox, further worsening liver toxicity. However, hepatoprotective effect and the role of antioxidants in naringenin are evident via alleviation of the liver oxidative stress markers and replenishing antioxidant [7] defense system dose dependently. Similar results are documented in earlier studies done by Renugadevi and Lee et al. [59,63], and results from the current study further validate the previous results. Therefore, prophylactic treatment of naringenin before Dox chemotherapy might be advantageous to cancer patients by mitigating toxicity produced by Dox treatment. The modulatory effect of naringenin could be attributed to its antioxidant potential. 

Reports demonstrate initiation of the inflammatory pathway as the basis of Dox-caused hepatotoxicity. Dox administration might activate pro-inflammatory supporting microenvironment with augmentation of pro-inflammatory cytokines such as TNF-α and IL-6 accompanied by inhibition of the anti-inflammatory supportive microenvironment. We examined the role of cytokines, which are low-molecular weight proteins that control functional aspects of cell like cell migration, cell survival, proliferation, immune cell activation, and apoptosis. They require a transcription factor for coding and instigation of inflammatory mediators which include IL-1β, Cox-2, NF-κB, and TNF-α. Additionally, other downstream cytokines such as IL-1β and IL-6 are stimulated by TNF-α, needed for inflammation [68]. In the present work, we evaluated NF-κB, Cox-2, TNF-α, IL-1β, PGE-2, TGF-β, and IL-6 in the Dox-treated group when compared with group I, and are indicative of organ inflammation and damage. Our reports are in concurrence with previous literature reports by Rehman et al., Song et al., Raso et al., and Karuppagounder et al. [6,16,26,42,69] (Table 4 and Figure 5). The free radical generation activates transcription factor, NF-κB. The transcription of pro-inflammatory cytokines which incites the inflammatory process of liver are activated by NF-κB. Naringenin treatment in Dox-treated groups reduced the levels of transcription factor and pro-inflammatory cytokine alleviating inflammation and cell death. It also decreases ROS, due to radical scavenging ability of hydroxyl groups at the 4th, 5th, and 6th positions of naringenin. The reduction in the levels of pro-inflammatory cytokines by the supplementation of naringenin is due to inhibitory action of naringenin on NF-κB in the present study. Similar findings have been obtained by Jayaraman et al., Rehman et al., and Lou et al. in their studies [12,24,27]. The phenolic structure of anthocyanins is responsible for their antioxidant activity, i.e., ability to scavenge reactive oxygen species (ROS) such as superoxide (O^2−^), singlet oxygen (‘O2), peroxide (ROO−), hydrogen peroxide (H_2_O_2_), and hydroxyl radical (OH) [39].

Reports by Kabel et al. and Sun et al. demonstrate that TGF-β1/STAT-3 signaling has an imperative role in the pathology of Dox-caused liver damage, and in particular, TGF-β1 pathway was demonstrated to mediate the toxic effects of Dox on various organs like heart, liver, and kidney. We obtained similar results as Dox upsurged TGF-β1 resulting in increased generation of pro-inflammatory cytokines in the present study [69,70] in the Dox-administered group as compared to the control group. However, naringenin administration regulated TGF-β1 levels and downstream molecules like IL-6, PGE2, and IL-1β levels at both the doses significantly which infers anti-inflammatory potential of naringenin. 

Our results are further supported by reports of Lou et al. and Rehman et al. in their studies. Histological results further validated our biochemical, serological, and immunohistochemical findings by showing the adverse effect of use of Dox in group II as compared to the control group animals as reported previously by Rashid et al. [11]. We observed that naringenin supplementation reversed the adverse effects of Dox administration and restored the normal histology. Similar results were found by Osama et al. in their study [57].

## 5. Conclusions

This study shows that naringenin has an advantageous role in the mitigation of Dox-mediated hepatotoxicity in rats possibly by scavenging free radicals produced by Dox prior to reaching DNA and triggering damage. Furthermore, naringenin might be stimulating DNA repair mechanisms. Hence, naringenin could be a potent chemopreventive agent to mitigate Dox-caused hepatotoxicity and simultaneous drug resistance. It may benefit patients receiving Dox in their treatment regimen after further mechanistic studies to avert secondary damage to them.

## Figures and Tables

**Table 1:** Tabular representation of experimental schedule.

Groups	Treatment
**Group I**	Animals received normal diet for 20 consecutive days.
**Group II**	A single intraperitoneal injection of Dox at the dose of 20 mg/kg body weight on 20th day along with normal diet for 20 consecutive days.
**Group III**	Animals were first treated with normal diet and naringenin (50 mg/kg body weight) for 20 days before the Dox treatment (as in group II).
**Group IV**	Animals were first treated with normal diet and naringenin (100 mg/kg body weight) for 20 days before the Dox treatment (as in group II).

All the animals were sacrificed on 21st day.

**Table 2:** Effect of treatment of naringenin on glutathione dependent and other antioxidant enzymes in different experimental groups.

	Group I	Group II	Group III	Group IV
**Reduced Glutathione** **(GSH; nmol mg** **protein)**	333.10 ± 15.1	146.12 ± 16.2 ***	192.32 ± 13.4 ^#^	307.42 ± 29.7 ^###^
**Oxidized Glutathione** **(GSSG; nmol mg** **protein)**	43.42 ± 3.02	89.32 ± 7.71 ***	64.34 ± 5.32 ^#^	46.03 ± 3.38 ^###^
**GPx (nmol/min/mg protein)**	190.39 ± 19.3	79.15 ± 7.07 ***	147.33 ± 12.1 ^##^	159.65 ± 11.2 ^##^
**GR (nmol min/min/mg protein)**	227.61 ± 19.6	101.31 ± 10.5 ***	165.09 ± 13.5 ^##^	210.20 ± 22.8 ^###^
**SOD (units/min/mg protein)**	14.93 ± 2.87	4.83 ± 0.71 ***	8.53 ± 0.82 ^#^	10.14 ± 1.32 ^###^
**Catalase (nmol H_2_O_2_ consumed/min/mg protein)**	9.41 ± 0.81	2.01 ± 0.11 ***	5.82 ± 0.51 ^###^	7.32 ± 0.64^###b^
**H_2_O_2_** **(nmol of H_2_O_2_** **/g tissue)**	192.1 ± 18.7	407.3 ± 19.2 ***	333.2 ± 26.1 ^#^	212.1 ± 19.5 ^###^

Group-I: normal control; group-II: doxorubicin (20 mg/kg bw); group-III: Dox rats treated with naringenin (50 mg/kg bw/day); and group-IV: Dox rats treated with naringenin (100 mg/kg bw/day). Data are represented as mean of six rats ± S.E.M.

**Table 3:** Effect of treatment of naringenin on ALT, AST, ALP, LDH, and total protein in different experimental groups.

Treatment Regimen	ALT (IU/L)	AST (IU/L)	ALP (IU/L)	Total Protein (IU/L)	LDH (nmol NADH oxidized/min/mg Protein)
**Group I**	67.73 ± 4.12	108.3 ± 9.9	103. 4± 9.11	6.01 ± 0.49	112.91 ± 15.3
**Group II**	119.4 ± 6.42 ***	300.6 ± 14.9 ***	201.3 ± 8.20 ***	3.13 ± 0.19 ***	302.23 ± 19.7 ***
**Group III**	99.21 ± 7.92 ^#^	210.2 ± 16.5 ^#^	172.2 ± 9.32 ^#^	3.87 ± 0.22 ^ns^	198.76 ± 25.9 ^#^
**Group IV**	80.31 ± 4.64 ^##^	151.1 ± 7.83 ^###^	115.9 ± 10.8 ^###^	5.01 ± 0.49 ^##^	151.03 ± 15.9 ^##^

Group-I: normal control; Group-II: doxorubicin (20 mg/kg bw); Group-III: Dox rats treated with naringenin (50 mg/kg bw/day); and Group-IV: Dox rats treated with naringenin (100 mg/kg bw/day). Data are represented as mean of six rats ± S.E.M.

**Table 4:** Effect of treatment of naringenin on inflammatory markers in different experimental groups.

	Group I	Group II	Group III	Group IV
**NFκ-B (pg/mL)**	901.62 ± 71.6	2341.10 ± 61.5 ***	1952.32 ± 38.2 ^#^	1102.32 ± 31.7 ^###^
**TNF-α (pg/mL)**	247.30 ± 17.8	552.18 ± 27.2 ***	321.01 ± 14.0 ^##^	269.11 ± 19.2 ^###^
**IL-1β (pg/mL)**	892.90 ± 41.4	1746.18 ± 61.0 ***	1485.44 ± 70.1 ^#^	901.14 ± 38.3 ^###^
**IL-6 (pg/mL)**	975.15 ± 80.2	2127.83 ± 57.1 ***	1763.20 ± 44.1 ^#^	1001.62 ± 67.7 ^###^
**TGF-β (pg/mL)**	259.18 ± 13.8	722.95 ± 42.1 ***	431.38 ± 21.4 ^##^	271.83 ± 31.8 ^###^
**PGE-2 (pg/mL)**	139.97 ± 14.4	527.36 ± 32.0 ***	154.11 ± 13.2 ^###^	146.02 ± 13.0 ^###^

Group-I: normal control; Group-II: doxorubicin (20 mg/kg bw); Group-III: Dox rats treated with naringenin (50 mg/kg bw/day); and Group-IV: Dox rats treated with naringenin (100 mg/kg bw/day). Data are represented as mean of six rats ± S.E.M.
